# LiBr‐Formic Acid Enables Lignocellulosic Biomass Fractionation within 10  Minutes

**DOI:** 10.1002/cssc.202501354

**Published:** 2025-10-03

**Authors:** Qi Bu, Shuzhen Ni, Zhaojiang Wang, Yingjuan Fu, Yongchao Zhang, Wenyang Xu

**Affiliations:** ^1^ State Key Laboratory of Green Papermaking and Resource Recycling Qilu University of Technology Shandong Academy of Sciences Jinan Shandong 250353 P. R. China; ^2^ Department of Sustainable and Bio‐Inspired Materials Max Planck Institute of Colloids and Interfaces Science Park Golm 14476 Potsdam Germany

**Keywords:** enzymatic hydrolysis, high‐purity component, lignocellulosic biomass, rapid fractionation, resource efficiency

## Abstract

Efficient biomass fractionation is essential for enhancing resource efficiency in the circular bioeconomy. However, achieving rapid, effective fractionation while minimizing the use of non‐recyclable chemicals remains challenging and also often yields low component purity. To circumvent these issues, an LiBr‐assisted formic acid (LB‐FA) system is developed that enables biomass fractionation within 10 min without auxiliary technologies. It is found that adding LiBr facilitates biomass swelling, improving liquid penetration and component‐removal efficiency. Moreover, LiBr promotes the dissociation of formic acid, enhancing the removal of hemicellulose and lignin. The LB‐FA system removed 96.0% of lignin and 99.6% of hemicellulose from poplar wood, yielding cellulose with 97.2% purity and lignin with 98.5% purity. The cellulose retained Iβ allomorphs, and enzymatic hydrolysis achieved over 90% glucose yield in 12 h. This strategy provides a rapid, sustainable route for producing high‐purity biomass components, facilitating bioethanol production and other sustainable material development applications.

## Graphical abstract

LiBr‐assisted formic acid fractionates lignocellulose in 10 min, removing 96.0% lignin and 99.6% hemicellulose to give high‐purity cellulose (97.2%, cellulose Iβ retained) and lignin (98.5%). The cellulose yields >90% glucose after 12 h of enzymatic hydrolysis.

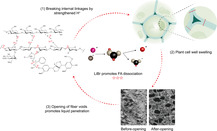

## 1 Introduction

Biomass, the most abundant renewable resource on Earth, is widely recognized as a sustainable alternative to fossil‐based energy and materials.^[^ ^1^ ^]^ Among its various forms, lignocellulosic biomass accounts for nearly half of global biomass volume, including wood, grasses, agricultural residues, and urban fibrous wastes.^[^ ^2^ ^]^ Despite its abundance, ≈89% of lignocellulosic biomass remains underutilized, primarily due to the lack of efficient fractionation technologies that can selectively separate its major components, that is, cellulose, hemicellulose, and lignin.^[^ ^3^ ^]^ In the plant cell wall, these biopolymers form a dense and recalcitrant matrix, held together by a complex bonding system including covalent bonds, hydrogen bonding networks, and van der Waals interactions.^[^ ^4^, ^5^ ^]^ This complex architecture confers structural rigidity but poses a significant challenge for biorefinery processing.

Conventional biomass fractionation approaches mostly integrate high temperatures and pressures to disrupt the plant cell wall, enhancing mass transfer and overcoming recalcitrance.^[^ ^6^ ^]^ While effective to some extent, these methods are energy‐intensive and often lead to undesirable side reactions, such as lignin condensation^[^ ^7^, ^8^ ^]^ and carbohydrate degradation, which compromise the purity and functionality of the resulting fractions.^[^ ^9^ ^]^ Therefore, developing low‐energy, high‐selectivity fractionation strategies is critical to improving the resource efficiency and sustainability of biomass conversion.

Organic acid‐based pretreatments have recently emerged as promising alternatives to conventional hydrothermal or alkaline methods.^[^ ^10^ ^]^ The acidic environment promotes selective cleavage of glycosidic linkages in hemicellulose, ether bonds in lignin, and lignin‐carbohydrate complexes (LCCs) while stabilizing cellulose via enhanced hydrogen bonding.^[^ ^10^, ^11^, ^12^ ^]^ Zhu et al. reported an acid hydrotropic fractionation strategy, where hydrotropic organic acids (e.g., *p*‐toluenesulfonic acid, maleic acid) can achieve efficient biomass fractionation under atmospheric pressure conditions. In those reported cases, the solvents used can be recovered from the processing waste liquid by simply diluting it with water, enabling the concentrated hydrophilic solvent to be reused.^[^ ^13^, ^14^, ^15^ ^]^ This highlights the potential of hydrotropic organic acids for energy‐efficient, selective, and rapid biomass fractionation under mild conditions. Formic acid, as a hydrotropic short‐chain organic acid, is widely used in biomass fractionation due to its excellent solubility of lignin.^[^ ^16^ ^]^ Formic acid can remove 85% of lignin at 100 °C, enabling effective biomass fractionation.^[^ ^17^ ^]^ However, mild reaction conditions also limit further improvements in fractionation efficiency. Although increasing reaction intensity and using auxiliary technologies can rapidly fractionate biomass components,^[^ ^18^, ^19^ ^]^ the applied harsh reaction conditions and the low scalability of auxiliary technologies still face limitations.

Increasing the availability of protons (H^+^) in the system is a promising strategy to further improve the efficiency and selectivity of formic acid‐based fractionation. Higher proton concentrations can enhance biomass deconstruction by promoting fiber swelling, increasing porosity, and accelerating lignin and hemicellulose removal.^[^ ^20^, ^21^ ^]^ We hypothesized that introducing an inexpensive inorganic additive into the formic acid system could facilitate H^+^ dissociation, thereby reducing energy input and achieving synergistic separation performance improvements. High‐concentration lithium bromide (LiBr)‐based molten salt developed by Pan et al. has been used for the biomass fractionation, various fractions could be produced, including polymeric hemicellulose, dissolving pulp,^[^ ^22^ ^]^ sugar monomers, furfural,^[^ ^23^ ^]^ nanocellulose,^[^ ^24^ ^]^ and uncondensed lignin.^[^ ^25^ ^]^ As a mild Lewis acid, LiBr offers a greener and less corrosive alternative to strong Brønsted acids, such as H_2_SO_4_ and HCl.^[^ ^26^, ^27^ ^]^ LiBr was reported to be capable of swelling fibers at ambient conditions and selectively dissolving hemicellulose.^[^ ^22^, ^23^, ^28^ ^]^ Inorganic salts like LiBr are also known to enhance solution acidity by promoting proton release from organic acids.^[^ ^29^, ^30^ ^]^ Therefore, LiBr is a compelling additive for enhancing formic acid‐based biomass fractionation.

Here, we report a LiBr‐assisted formic acid (LB‐FA) system that enables ultrafast and energy‐efficient fractionation of lignocellulosic biomass. Contrary to ab initio molecular dynamics simulations suggesting that LiBr may suppress H^+^ dissociation at room temperature with a formic acid: LiBr molar ratio of 1:3 at 27 °C,^[^ ^31^ ^]^ our experimental results demonstrate that, at elevated temperatures and low LiBr concentrations (formic acid: LiBr molar ratio of 33:1 at 120 °C), LiBr enhances deprotonation of formic acid, facilitating H^+^ release. In our study, we systematically optimized the LB‐FA system by tuning key parameters, including temperature, reaction time, and LiBr dosage, and evaluated its fractionation performance on poplar biomass. Mechanistic investigations revealed that the LB‐FA system promotes rapid fiber swelling and efficient removal of hemicellulose and lignin, yielding high‐purity cellulose suitable for enzymatic saccharification. This work establishes a new, scalable platform for selective biomass deconstruction and represents a significant advance in developing sustainable biorefinery technologies.

## 2 Results and Discussion

### 2.1 Fractionation Performance Using the LB‐FA System

**Figure 1:**
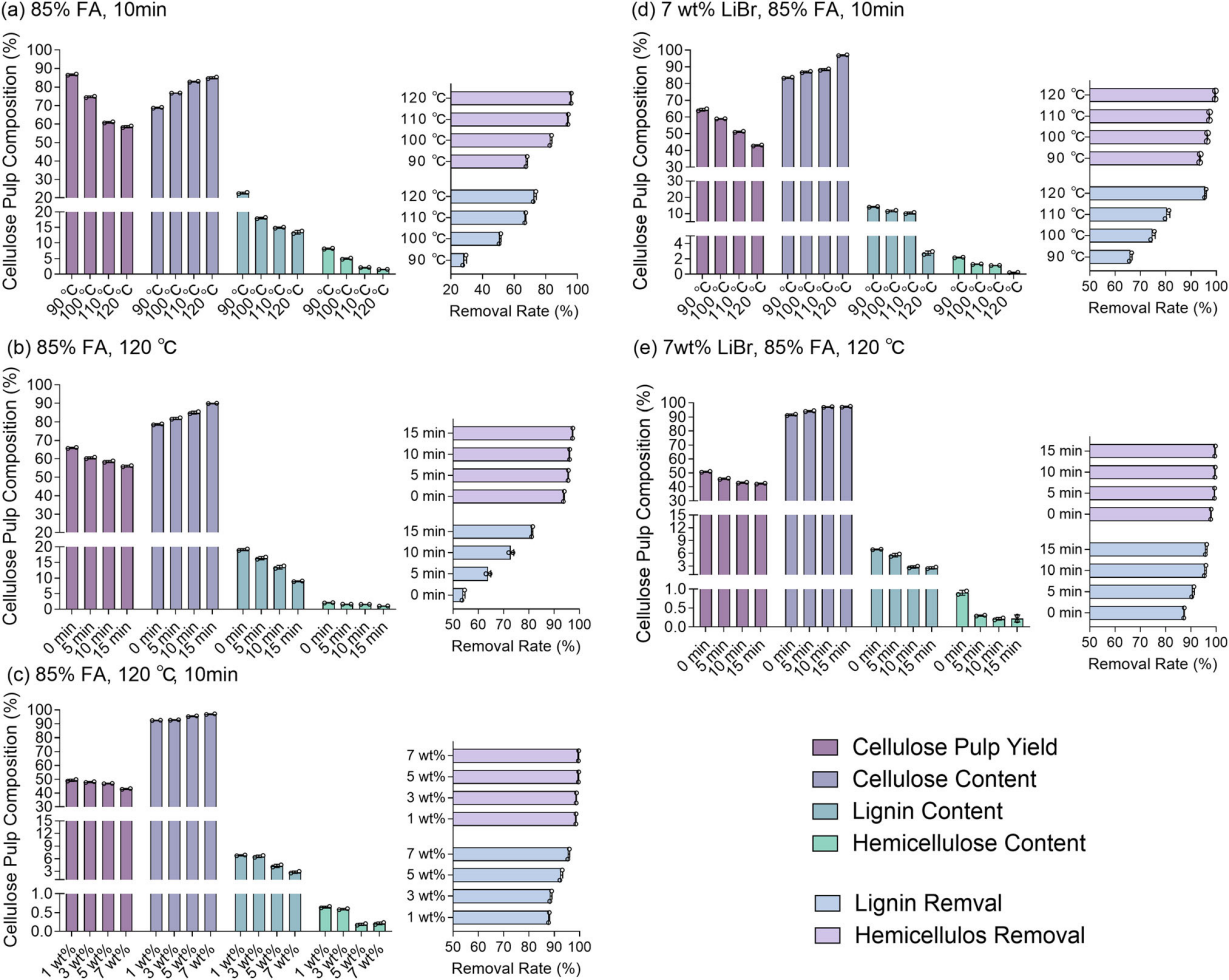
Components of cellulose pulp obtained from various fractionation conditions. a,b) show the content of three components in cellulose pulp using the FA fractionation systems without the addition of LiBr under different reaction temperatures and reaction times, respectively; c–e) show the compositions in cellulose pulp using LB‐FA fractionation systems under different dosage of LiBr, reaction temperatures, and reaction time, respectively. The 0 min in (e) shows the composition at the warming phase (The process from room temperature to target temperature). The 85% formic acid concentration is the conventional concentration in industrial production. Note: the yield of cellulose pulp is calculated relative to the mass of the raw material; the composition ratio of cellulose, lignin, and hemicellulose in pulp is calculated relative to the mass of cellulose pulp; the removal rate is calculated based on the mass of that content in the raw material (Formula S1, Supporting Information).

**Figure** **1** shows the compositional change of LB‐FA fractionation of Poplar wood with reference to the solely FA system (Figure 1a,b) at various reaction conditions, including reaction temperature, treatment duration, and different addition amounts of LiBr. The fractionation using the FA system across different temperatures showed limited fractionation efficacy (Figure 1a). In contrast, as shown in Figure 1d, the LB‐FA fractionation system showed improved fractionation efficacy with the addition of LiBr. The content of cellulose in pulp reached 97.2% due to the efficient removal of lignin and hemicellulose at the conditions of using 85.0% FA, 7 wt% LiBr, 120 °C, 10 min, compared to that of 85.0% FA without LiBr. Correspondingly, lignin and hemicellulose contents in pulp were reduced from 14.4% to 2.6% and decreased from 2.2% to 0.2%, respectively. At an optimized temperature of 120 °C, lignin removal reached 96.0%, which is 22.3% higher than that of the FA system at the same temperature. In addition, the addition of LiBr could shorten the treatment time, as revealed by Figure 1b,e. Notably, the LB‐FA fractionation system achieved 87.4% lignin removal in 0 min, surpassing the 15‐minute treatment of FA system (81.5%). The fractionation efficacy plateaued at the treatment of 10 min, where extending to 15 min barely increased the lignin removal to 96.3% compared to that of 10 min, reaching 96.0%. As shown in Figure 1c, the main component‐removal rate increased with the increase of LiBr concentration, where the addition of 1 wt% LiBr could already facilitate 98.5% hemicellulose removal. The removal rates of lignin (96.0%) and hemicellulose (99.6%) leveled off when LiBr was increased to 7 wt%. To avoid further biomass degradation as induced by high dosage of LiBr,^[^ ^32^ ^]^ we chose 7 wt% for the following studies.

**Figure 2:**
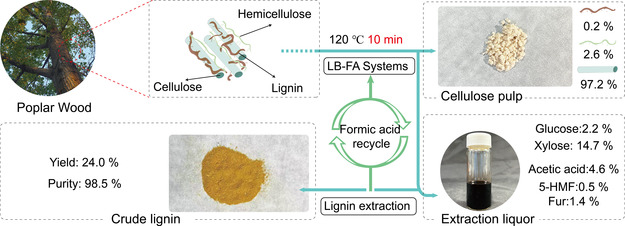
Schematic summary of step‐wise compositional analysis of the major components and poplar wood fractionation using the LB‐FA system. Note: 5‐Hydroxymethylfurfural (5‐HMF); Furfural (Fur).

The optimal conditions of 120 °C, 10 min, and 7 wt% LiBr was determined by comparing three single‐factor reaction conditions and summarized in **Figure** **2**. Under these conditions. The LB‐FA fractionation system shows the highest cellulose pulp purity (97.2%), lignin removal rate (96.0%), and hemicellulose removal rate (99.6%). This concluded that the LB‐FA system can effectively fractionate the major components within 10 min. This might be attributed to the fact that the addition of LiBr can effectively swell and dissolve cellulose,^[^ ^28^, ^33^ ^]^ which will be discussed in a later session; meanwhile, it can also promote hemicellulose hydrolysis.

### 2.2 Compositional Analysis of the Filtrate Liquids

**Figure 3:**
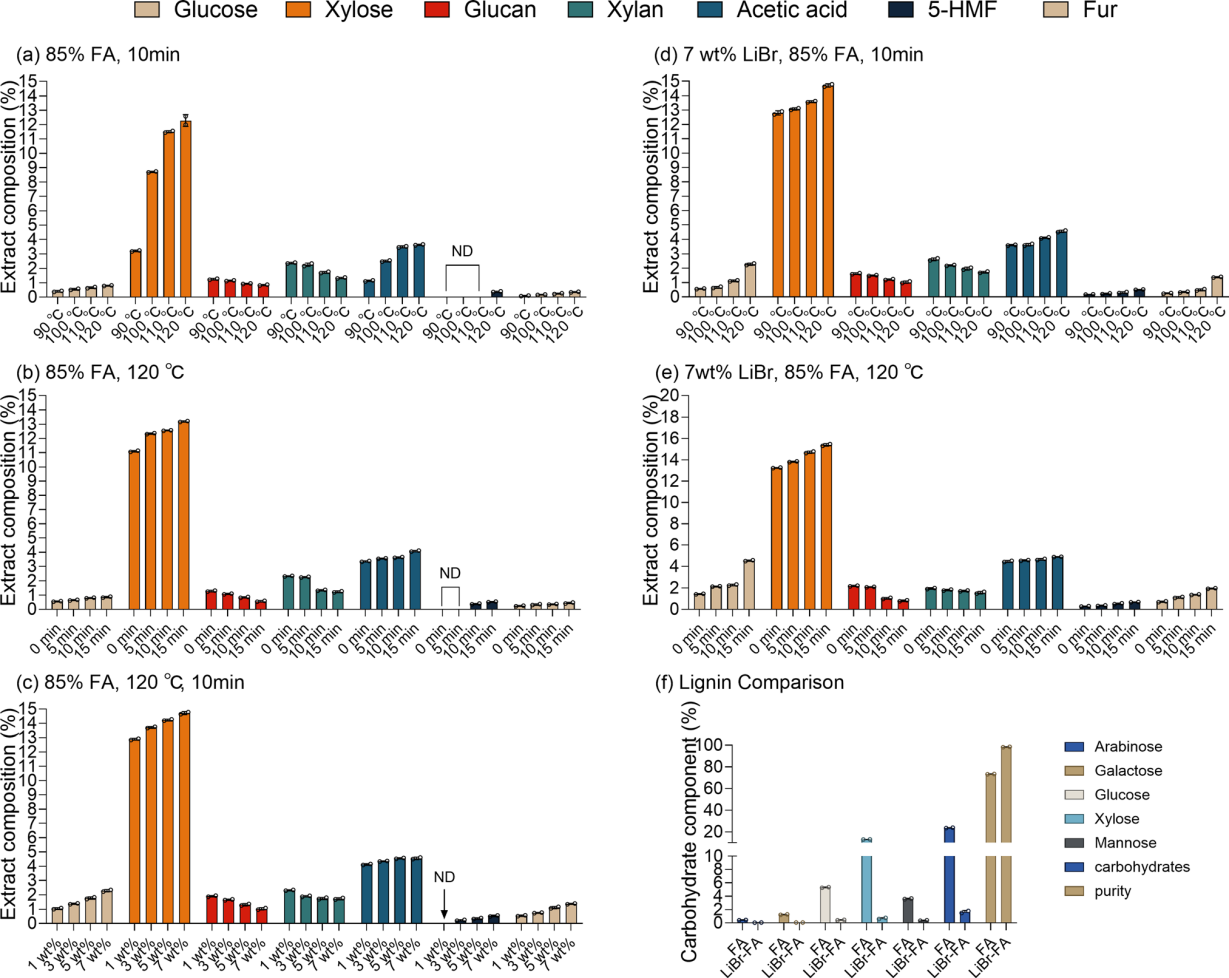
Carbohydrate content in the extract solutions obtained from various conditions. a,b) show the content of carbohydrates in extract solution using FA fractionation without adding LiBr under different reaction temperatures and reaction times, respectively; c–e) show the content of carbohydrates in extract solution using LB‐FA fractionation under different dosage of LiBr, reaction temperatures and reaction times, respectively. The 0 min in (e) indicates the onset of the warming phase. f) show the purity and carbohydrate of two lignins that LB‐FA lignin under optimal conditions of 120 °C, 10 min, and 7 wt% LiBr, and FA lignin under conditions of 120 °C, 10 min. Note: The content of monosaccharides, glycans, and degradation products is calculated relative to the mass of the raw material; the content of carbohydrates in lignin is calculated relative to the mass of lignin in the extract solution; Lignin purity calculated as 100% subtracting carbohydrate content (%); ND stands for not detected.

The content of carbohydrates in the extract solution associated with hemicellulose hydrolysis in the LB‐FA fractionation system is summarized in **Figure** **3**. In the organic acid fractionation, hemicellulose is prone to being first hydrolyzed and converted to monosaccharides such as glucose and xylose. Further degraded products such as furfural,^[^ ^34^ ^]^ 5‐Hydroxymethylfurfural (5‐HMF), and acetic acid,^[^ ^35^, ^36^ ^]^ from the degradation of xylose and glucose, respectively, were also found in the extract.^[^ ^37^, ^38^ ^]^

The composition of carbohydrates in the FA and LB‐FA fractionation systems’ extract solutions at different temperatures are shown in Figure 3a,d, respectively. The contents of xylan and glucan in the two fractionation systems decreased, while the corresponding xylose and glucose contents increased with increasing temperature. This is because xylan and glucan were hydrolyzed to xylose and glucose due to the enhancement of catalytic hydrolysis with increasing temperature. The hemicellulose hydrolysis efficiency of the LB‐FA system was higher than that of the conventional FA system under the optimized conditions at 120 °C. Notably, the xylose content of the LB‐FA extract has reached 12.7% under the mild condition of 90 °C, which is higher than that of the FA system at 120 °C (12.0%). This demonstrated the efficient hemicellulose hydrolysis of the LB‐FA system under mild temperature conditions. As shown in Figure 3b,e, with increasing reaction time, the LB‐FA system continued to demonstrate an advantage in hydrolysis, with the xylose content increasing from 13.2% to 15.3%, while the FA system increased from 11.1% to 13.2%. The xylose content peaked at 14.7% when LiBr was added up to 7 wt% (Figure 3c).

In addition, the LB‐FA system promoted the formation of high‐value‐added products from hemicellulose‐derived sugars. Analysis of the extract solution (Figure 3a–e) revealed significantly higher degradation product yields than the FA system, with furfural emerging as the predominant compound. Under optimal conditions (120 °C, 7 wt% LiBr, 10 min), the furfural content reached 1.4%, approximately five times higher than that obtained with solely formic acid (≈0.3%). This result highlights the distinctive advantage of the LB‐FA system in enabling selective xylose conversion into furfural, offering added value alongside efficient biomass fractionation.

Lastly, the LB‐FA system also facilitated the cleavage of lignin–carbohydrate complexes (LCCs). It is well established that covalent linkages between lignin and carbohydrates form LCCs, which hinder lignin purification and limit its valorization potential.^[^ ^39^ ^]^ As shown in Figure 3f, the LB‐FA system achieved a lignin purity of 98.5%, with only 1.5% residual carbohydrates compared to solely formic acid hydrolysis (76.5% purity, 23.5% carbohydrates). This can be attributed to the effective cleavage of LCC linkages, enabled by the synergistic catalytic action of LiBr, which promotes dissociation of chemical bonds between lignin and carbohydrates. Collectively, the LB‐FA system not only accelerates hemicellulose hydrolysis but also selectively disrupts LCC structures, yielding high‐purity lignin suitable for downstream conversion into high‐value products.

### 2.3 Potential Mechanism of Effective Fractionation of LB‐FA

#### 2.3.1 Characteristic Analyzes of Treated Wood Using LB‐FA

**Figure 4:**
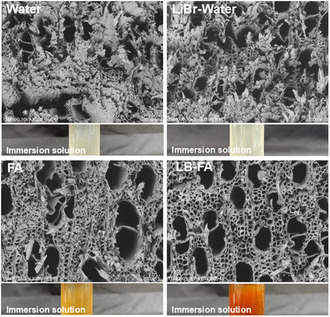
Cross section of poplar wood slices after being subjected to different solutions including water, water with LiBr, formic acid without and with LiBr at room temperature for 12 h with below corresponding solution after immersion.

To gain insight into the role of LiBr in the LB‐FA fractionation system, we investigated its effect by immersing poplar wood in various solvents at room temperature and analyzing the resulting morphological changes. Cross‐sectional images (**Figure** **4**) show that samples soaked in water retained a densely packed structure, with only a few open fiber pores, indicating minimal swelling. Adding LiBr to water led to slightly more pore opening, while formic acid induced noticeable swelling and pore formation. Strikingly, the LB‐FA system exhibited the most pronounced fiber swelling, with a visibly expanded porous structure. This enhanced swelling increases the fiber's specific surface area, improving the accessibility of liquid reagents and enabling a more efficient component separation.

In addition to morphological changes, the LB‐FA solution showed a dramatic color change among the four treatments, suggesting higher dissolution of biomass components. HPLC analysis (Table S3, Supporting Information) confirmed a markedly higher carbohydrate concentration in the LB‐FA immersion liquor than that of formic acid alone, further demonstrating the superior fractionation capability of the LB‐FA system. These results collectively highlight the synergistic effect of LiBr and formic acid in promoting fiber swelling and facilitating efficient biomass deconstruction.

LiBr is most often employed in a high‐concentration, molten hydrated state (MSH) to depolymerize cellulose or hemicellulose into high‐value products, leveraging MSH's ability to swell and dissolve cellulose at temperatures above 100 °C.^[^ ^22^, ^28^, ^40^ ^]^ In particular, a 60 wt% LiBr system removes 95.25% of hemicellulose at 80 °C in 5 h.^[^ ^22^ ^]^ Under acidic conditions, 84.55% cellulose degradation is achieved at 100 °C in 0.5 h.^[^ ^40^ ^]^ Raising the temperature to 110 °C leads to complete degradation of both cellulose and hemicellulose.^[^ ^28^ ^]^ These results indicate that LiBr's swelling/dissolution efficiency is markedly enhanced by acidity and elevated temperature. Conversely, Lara‐Serrano et al.^[^ ^41^ ^]^ showed that greater hydration (i.e., lower LiBr concentration) limits cellulose degradation, implying that LiBr concentration governs whether the system primarily swells or depolymerizes fibers. Practically, acidity, temperature, and LiBr hydration should be tuned to regulate swelling versus degradation. In this study, soaking poplar wood in a 7 wt% LiBr–formic acid (LiBr–FA) system for 12 h at room temperature produced substantial fiber swelling, indicating that even with ≈88% less LiBr than the conventional 60 wt% system, effective swelling persists under strongly acidic conditions. We attribute this to two factors: 1) delignification and hemicellulose removal, which enlarge pore volume and 2) the intrinsic swelling action of highly hydrated LiBr, which acts as a swelling agent rather than a dissolution medium. Thus, the LiBr–formic acid (LiBr–FA) system promotes pronounced fiber swelling at room temperature, demonstrating high efficacy under mild conditions. From a scale‐up perspective, replacing ≈88% of LiBr with recyclable formic acid preserves performance, reduces costs, and lowers environmental burden, in line with sustainable development goals.

#### 2.3.2 LiBr‐Formic Acid Synergistic Hydrolysis

As shown in **Table** **1**, adding LiBr produced a clear, concentration‐dependent decrease in the measured pH of both aqueous and formic‐acid (FA) media, consistent with an increase in proton activity. Certain inorganic salts can enhance apparent acidity via hydrolysis, coordination, or ionic‐strength effects that favor acid dissociation and elevate proton activity.^[^ ^29^, ^30^ ^]^ On this basis, we hypothesized that LiBr similarly promotes FA dissociation, thereby increasing H^+^ activity and improving component‐removal efficiency. To probe this synergy, we measured pH across LiBr concentrations. Because 85% FA lies outside the reliable range of standard glass electrodes, it was diluted with water (2:3 v/v) to an initial pH of 1.30. In water, 7 wt% LiBr lowered the pH from 6.97 to 4.19, indicating that LiBr alone can increase apparent acidity. In the FA, increasing LiBr to 12 wt% further reduced pH from 1.30 to 0.61, demonstrating a strong synergistic effect between LiBr and FA. We attribute this to Li^+^acting as a Lewis acid, coordinating with formate (HCOO^−^) and shifting the FA dissociation equilibrium toward greater H^+^ release, together with ionic‐strength effects on activity coefficients. This is analogous to coordination‐enhanced proton activity, which has been reported for metal salts such as FeCl_3_.^[^ ^30^ ^]^ Combined with scanning electron microscopy (SEM) evidence of pronounced fiber swelling, these results support a dual‐synergy mechanism in the LiBr–FA system: 1) structural disruption and 2) acidity enhancement, both driving efficient biomass fractionation.

**Table 1:** The pH values of formic acid with addition of different amount of LiBr.

Sample	LiBr dosage	pH
HPLC Water	0 wt%	6.97 ± 0.18
HPLC Water	7 wt%	4.19 ± 0.08
Formic acid	0 wt%	1.30 ± 0.03
Formic acid	1 wt%	1.14 ± 0.01
Formic acid	3 wt%	1.07 ± 0.02
Formic acid	5 wt%	0.91 ± 0.04
Formic acid	7 wt%	0.76 ± 0.05
Formic acid	10 wt%	0.68 ± 0.01
Formic acid	12 wt%	0.61 ± 0.04

#### 2.3.3 NMR Analysis

**Figure 5:**
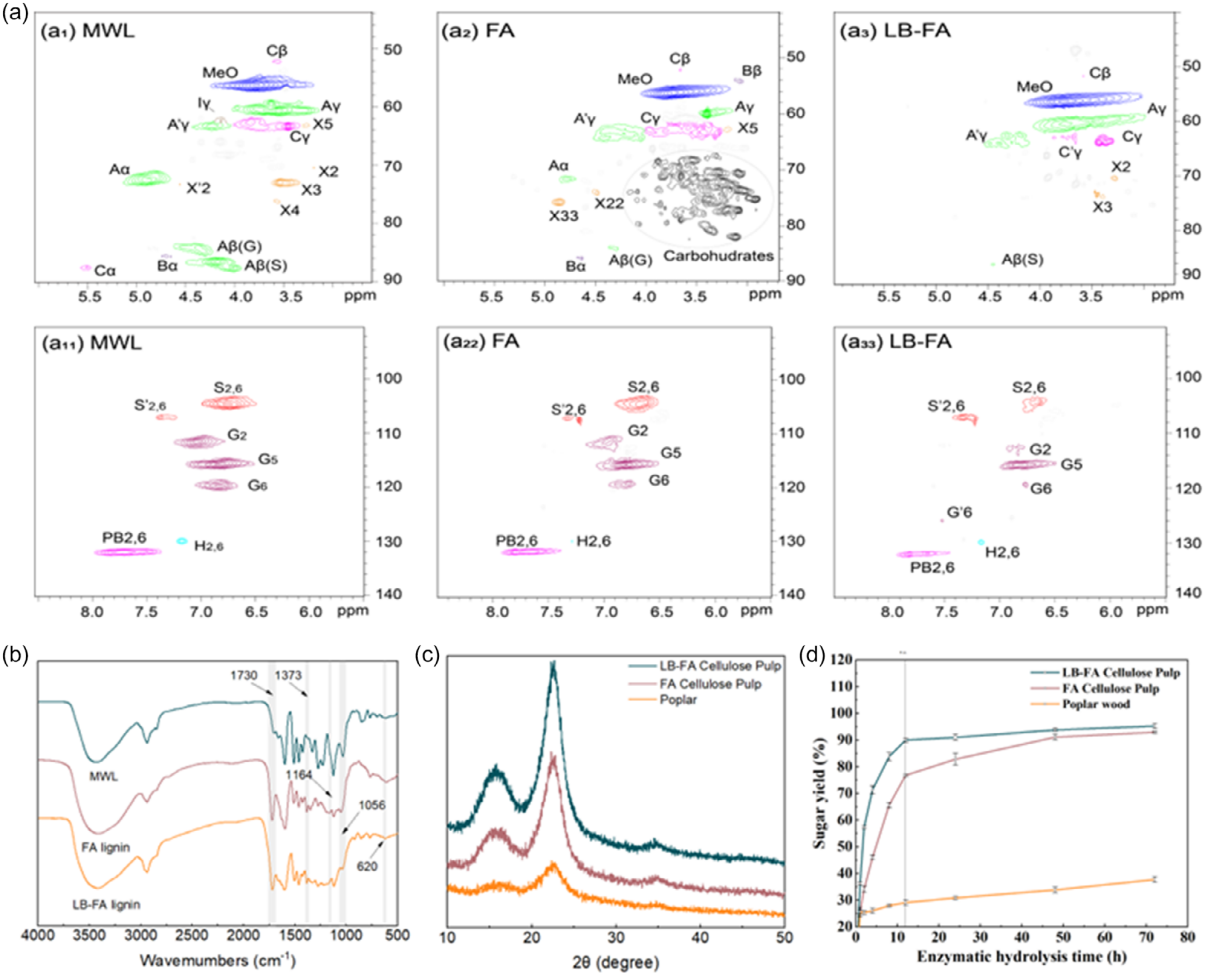
a) show the 2D HSQC NMR spectra of lignin; a_1_), a_2_), and a_3_) show the chemical signals of MWL, FA, and LB‐FA lignin in the fatty region, respectively; a_11_), a_22_), and a_33_) show the chemical signals of show the chemical signals of MWL, FA, and LB‐FA lignin in the aromatic region, respectively. b) Infrared spectra of MWL, and isolated lignin from formic acid and LiBr‐FA fractionation process; c) XRD patten of original Poplar wood, FA fractionaed cellulose pulp and LB‐FA cellulose pulps; d) Glucose conversion of cellulose pulp of original Poplar wood, after treatments using only formic (FA) and LiBr ‐ assisted formic acid fractionation (LB‐FA).

To elucidate the delignification mechanism of poplar in the LB–FA system, we conducted a systematic structural analysis of isolated lignins. 2D HSQC NMR (**Figure** **5**a) was used to track linkage distributions and their evolution during LB–FA fractionation. Cross‐peak assignments (Table S4, Supporting Information) and the primary substructures (Figure S1, Supporting Information) were established by comparison with the literature.^[^ ^42^ ^]^ Semi‐quantitative integration of HSQC contours provided the relative abundances of the principal interunit linkages, thereby evidencing lignin structural changes (**Table** **2**). In parallel, quantitative ^31^ P NMR was employed to determine aliphatic hydroxyl, phenolic hydroxyl, and carboxylic acid contents, where chemical‐shift assignments followed literature precedents,^[^ ^43^ ^]^ and results are summarized in **Table** **3**. Complementary molecular‐weight data for milled wood lignin (MWL)‐, FA‐, and LB–FA‐derived lignins are reported in Table S5, Supporting Information.

**Table 2:** NMR analysis results of 2D HSQC.

Lignin	*β*—O—4	*β*‐*β*	*β*‐5	*β*‐1	S/G ratio
MWL	83.1	4.3	2.9	N/D	1.4
FA	45.0	2.1	N/D	N/D	2.8
LB‐FA	N/D	N/D	N/D	N/D	2.5

Values in the table are based on 2D HSQC NMR, and the results are presented as the amount per 100 Aromatic unit.

**Table 3:** Phenolic hydroxyl content of MWL, FA and LB‐FA lignin.

Lignin	A‐OH	S‐OH	G‐OH	H‐OH	Sc‐OH	Ca	T‐OH
MWL	4.49	0.21	0.70	0.61	0.01	0.05	1.53
FA	5.48	0.43	0.17	0.16	0.1	0.29	0.76
LB‐FA	0.58	0.97	0.37	0.22	0.03	0.12	1.56

A‐OH: aliphatic hydroxyl group; S‐OH: S‐type phenolic hydroxyl group; H‐OH: H‐type phenolic hydroxyl group; Sc‐OH: S‐type condensed phenolic hydroxyl group; Ca: carboxylic acid; T‐OH: total phenolic hydroxyl group.

The side‐chain (aliphatic) region of the HSQC spectra for MWL (Figure 5a_1_), FA lignin (Figure 5a_2_), and LB‐FA lignin (Figure 5a_3_) shows the characteristic cross‐signals of methoxy groups (–OCH_3_), β—O—4 aryl ethers (A), and phenylcoumarans (B). Relative to MWL, the β—O—4 and β—β linkages are markedly depleted in the LB‐FA lignin, evidencing cleavage of primary interunit bonds during delignification. Notably, whereas FA lignin still contains 45.0% β—O—4 linkages, the β—O—4 signal is absent in LB‐FA lignin, confirming that β—O—4 scission is the dominant reaction in LB‐FA depolymerization. Distinct carbohydrate cross‐signals appear in the aliphatic region of FA lignin but are negligible in LB‐FA lignin. This trend is consistent with FTIR results (Figure 5b) and lignin–carbohydrate analyzes, where carbohydrate bands at 1730, 1373, 1164, and 1056 cm^−^^1^ are observed for FA lignin but are substantially weaker for LB‐FA lignin.^[^ ^43^, ^44^, ^45^ ^]^ Together, these observations indicate that the LB‐FA system disrupts nearly all LCC linkages within 10 min.

In addition, weak absorption near ≈620 cm^−^^1^ is observed in LB‐FA samples, assignable to C—Br stretching in lignin side chains (typically 750–550 cm^−^^1^).^[^ ^46^ ^]^ Oxygen‐combustion ion chromatography of LB‐FA lignin shows a Br content of 39.4 mg g^−^^1^ (Table S6, Supporting Information), suggesting bromination during depolymerization, in line with Li et al.^[^ ^28^, ^47^ ^]^ The S, G, and H unit signals (Figure 5a1–a3) diminish progressively from MWL to FA to LB‐FA lignin, indicating net degradation accompanied by some condensation during fractionation.^[^ ^15^ ^]^ The S/G ratios for MWL, FA lignin, and LB‐FA lignin are 1.4, 2.8, and 2.5, respectively, implying preferential degradation of G‐type units during rapid delignification, which is consistent with the higher reactivity of guaiacyl units under acidic conditions.

Compared to MWL, both FA and LB‐FA lignins show higher carboxyl group contents, indicating that partial oxidation accompanies component separation. Compared with FA lignin, LB‐FA lignin contains fewer aliphatic hydroxyls and a higher total phenolic‐OH content, further evidencing scission of β—O—4 linkages during LB‐FA fractionation.^[^ ^42^, ^48^ ^]^ In addition, the *M*_w_ of LB‐FA lignin (8,565) is lower than that of FA lignin (9,327), indicating that LiBr addition suppresses unwanted condensation reactions. Notably, *M*_n_ drops from 4,699 (FA) to 796 (LB‐FA), consistent with severe depolymerization and the loss of dominant interunit linkages. The resulting “high‐*M*_*w*_/low‐*M*_*n*_” profile yields a broad dispersity (Đ = *M*_*w*_/*M*_*n*_ = 10.8), far exceeding that of MWL and FA lignin, indicative of a heterogeneous mixture of macromolecular fragments and oligomers. ^31^ P NMR shows the condensed syringyl phenolic‐OH (S_c–OH) content in LB‐FA lignin is only 0.03, substantially lower than in FA lignin (0.10), signifying minimal condensation. We therefore attribute the extreme Đ to competing processes: rapid acidolytic β—O—4 scission generating low‐Mn fragments, coupled with slight acid‐catalyzed condensation of transient carbocations that produce high‐*M*_*w*_ residues. Overall, the LB‐FA system rapidly and selectively cleaves β—O—4 bonds within 10 min while suppressing condensation, affording highly reactive, high‐purity lignin.

#### 2.3.4 XRD and Enzymatic Hydrolysis Analysis

X‐ray diffraction (XRD) of LB‐FA and FA cellulose pulp in optimal conditions and untreated poplar powder are shown in Figure 5c, and the crystallinity index (*CrI*) and DP data are shown in Table S2, Supporting Information. Compared to the untreated poplar, the diffraction peaks of LB‐FA and FA cellulose pulp became distinct due to the progressive elimination of amorphous components.^[^ ^49^ ^]^ Under the optimal conditions (120 °C for 10 min), the crystallinity index (*CrI*) of FA and LB‐FA cellulose pulp was 66.0% and 69.4%, respectively, and the crystallinity of LB‐FA increased 3.4%. This suggests that the swelling of the LB‐FA system occurs in the wood matrix without swelling crystalline region of cellulose. Thus, the cellulose of LB‐FA still maintains the structure of cellulose I*β*. The diffraction peaks of the fibers at 2*θ* angles of 15.5°, 22.5°, and 34.6° were attributed to the typical characteristic peaks of cellulose I*β.* ^[^ ^50^ ^]^ In addition, compared to the DP of FA, the DP of LB‐FA was slightly lower than that of FA, which was 682 and 674, respectively.

The enzymatic hydrolysis of cellulose is often limited by the inherent recalcitrance of lignocellulosic biomass, where the compact structure and close association of cellulose with lignin and hemicellulose significantly reduce enzyme accessibility.^[^ ^51^, ^52^ ^]^ To address this, traditional strategies such as acid–base delignification, increased cellulase loading, and the addition of nonionic surfactants have been employed to enhance glucose conversion efficiency.^[^ ^53^, ^54^ ^]^ While effective, these approaches entail higher processing costs and resource consumption. In contrast, the LB‐FA fractionation system enables efficient cellulose hydrolysis without requiring supplementary treatments. As shown in Figure 5d, the LB‐FA‐derived cellulose pulp achieved a glucose conversion efficiency of 95.9% for 72 h of enzymatic hydrolysis at an enzyme loading of 20 FPU g^−1^, owing to the near‐complete removal of hemicellulose and lignin during pretreatment. Under identical conditions, untreated poplar and FA‐treated cellulose pulp reached only 38.4% and 93.2%, respectively. Remarkably, LB‐FA pulp achieved a conversion rate of 90.4% within 12 h, significantly reducing the enzymatic hydrolysis time compared to existing fractionation technologies (Table S7, Supporting Information). These results underscore the effectiveness of the LB‐FA system in enhancing cellulose digestibility, offering a streamlined and cost‐efficient pathway for high‐yield glucose production.

#### 2.3.5 Evaluation of the Reuse of LB‐FA

Reusability of the LB–FA system is essential for practical deployment and economic viability. Unlike high‐concentration LiBr processes (>60 wt%), which require energy‐intensive multistage evaporation and antisolvent precipitation to recover LiBr, the LB–FA system uses formic acid as the primary solvent and only a low LiBr loading (7 wt%) as an auxiliary. Given LiBr's low usage, cost, and environmental compatibility, complete recovery is not primarily considered, because trace residues do not measurably affect performance. Accordingly, our recycling study focuses on formic acid. Formic acid was recovered at 65 °C by rotary evaporation and detected by high‐performance liquid chromatography (HPLC). No hemicellulose sugars were detected, indicating that no sugar accumulation occurred during the cycle. The remaining solids (a hemicellulose‐lignin mixture) can be handled in two ways: 1) separation and recovery: an antisolvent strategy (tetrahydrofuran‐ethyl ether) separates and recovers lignin and hemicellulose (see Materials and Methods) and 2) Direct utilization: the recovered hemicellulose‐lignin mixture (98.5% lignin purity; Figure 3f) can serve directly as feedstock for lignin‐based adhesives, obviating further purification; this route will be explored in future work. As summarized in **Table** **4**, high fractionation performance was maintained over three consecutive recycles. Even after the third reuse, cellulose purity remained >95%, while lignin and hemicellulose removal efficiencies exceeded 93% and 98%, respectively. Filtrate sugar analyzes across cycles showed <1% variation in xylose, indicating excellent process stability and repeatability. Overall, the LB–FA system provides a robust, reusable fractionation platform that reduces processing costs and enhances the sustainability and industrial viability of biorefining operations.

**Table 4:** Recovery of LB‐FA and main component content in cellulose pulp.

Sample	Cellulose content [%]	Hemicellulose removal [%]	Lignin removal [%]	Glucose content [%]	Xylose content [%]	Glucan content [%]	Xylan content [%]
LB‐FA fresh	97.2	99.6	96.0	2.3	14.6	1.1	1.8
1st reuse	96.3	98.6	94.8	2.1	13.7	1.1	1.5
2nd reuse	95.6	98.4	94.5	2.1	14.1	1.2	1.6
3rd reuse	95.0	98.3	93.4	2.3	13.6	1.2	1.5

The composition ratio of cellulose in pulp is calculated relative to the mass of cellulose pulp; the removal rate is calculated based on the mass of that content in the raw material. The content of glucose, xylose, glucan, and xylan in filtrate liquids is calculated based on the raw material.

## 3 Conclusions

We have developed a highly efficient LB‐FA fractionation strategy for the selective separation of lignocellulosic biomass components under mild conditions. Incorporating LiBr enhanced H^+^ dissociation from formic acid and promoted fiber swelling, facilitating mass transfer and enabling a dual synergistic mechanism that significantly improved fractionation performance. Under optimized conditions (120 °C, 7 wt% LiBr, 10 min), the process yielded high‐purity cellulose pulp (97.2%) while preserving the native cellulose I structure, alongside near‐complete removal of lignin (96.0%) and hemicellulose (99.6%). The recovered lignin displayed high purity (98.5%), and minimal sugar degradation was observed, with 14.6% of xylose retained in the liquor. The resulting cellulose exhibited excellent enzymatic digestibility, achieving over 90% glucose yield within 12 h.

These results establish LB‐FA as a low‐energy, high‐selectivity platform for lignocellulose fractionation, offering high‐quality intermediates for downstream conversion into bio‐based chemicals and materials. This work contributes a scalable and sustainable pathway toward next‐generation biorefining.

## 4 Experimental Section

4.1

4.1.1

##### Materials

Poplar powder of 40–60 mesh from Suqian, Jiangsu Province, China. The chemical composition of the poplar was characterized according to the standards of the National Renewable Energy Laboratory (NREL). It contains 42.19% of cellulose, 23.73% of hemicellulose, 29.48% of lignin, 3.48% of ethanol‐toluene extract, and 1.12% of ash. LiBr and KBr were purchased from Shanghai Macklin Biochemical Co., Ltd. Formic acid (85%) was purchased from Jinan Qiguang Science and Trade Co., Ltd. Tetrahydrofuran was purchased from Tianjin Fuyu Fine Chemical Co., Ltd. Ethyl ether was purchased from Yantai Far East Fine Chemical Co., Ltd. Na_2_CO_3_ and NaHCO_3_ were purchased from Thermo Fisher Scientific (USA). Copper ethylenediamine purchased from China Pulp and Paper Research Institute Co., Ltd. The cellulase enzyme (Cellic CTec3) was purchased from Novozymes (China) Biotechnology Co., Ltd.

##### LB‐FA Fractionation

Poplar powder and formic acid were mixed in a pressure‐resistant reactor at a solid‐to‐liquid ratio of 1:10 w/v, with 1–7 wt% LiBr added. The mixture was treated at various temperatures. Following the fractionation, the resulting cellulose pulp and extraction solution were separated using a Büchner funnel under vacuum filtration. The cellulose pulp was washed sequentially with 50 mL of 90 °C formic acid and then rinsed three times with deionized water. The pulp was dried in an electrothermal blast drying oven at 105 °C for 4 h and stored at 4 °C for further analysis.

The combined filtrate and washings were subjected to rotary evaporation to recover formic acid. After acid recovery, the residue was mixed with tetrahydrofuran (THF) in a 1:3 volume ratio at 5000 rpm for 5 min. Lignin dissolves in the supernatant, while hemicellulose is separated as an insoluble residue. Transfer approximately one‐third of the supernatant and replenish with fresh THF. This extraction‐evaporation process was repeated until the supernatant became colorless. Transferred supernatant using a rotary evaporator to concentrate the supernatant and recover THF. The final concentrated solution was mixed with ethyl ether at a volume ratio of 1:10 to precipitate lignin, which was collected as the lignin fraction. Recovery of ethyl ether using a rotary evaporator. For comparison, MWL was prepared according to the method described by Björkman.^[^ ^55^ ^]^

(1) $$\text{Removal rate}\left(\%\right)=\left[1-\left(\frac{a\times m_{1}}{m_{2}}\right)\right]\times 100\%$$

where *a* is the content of hemicellulose or lignin in cellulose pulp, *m*_1_ is the quality of cellulose pulp and *m*_2_ is the quality of hemicellulose or lignin in untreated poplar wood.

##### XRD Analysis

The crystallinity of the cellulose pulp was analyzed using an X'pert PROMPD diffractometer (SmartLab SE, Japan). The irradiation source, consisting of Cu‐Kα rays, was used at a voltage of 40 kV and a current of 200 mA. The scan was performed at a rate of 10°/min, covering the diffraction angle (2*θ*) ranging from 10.0 to 60.0°. The crystallinity index (*CrI*) was assessed using a method referenced in the literature.^[^ ^56^, ^57^ ^]^ It was computed based on the relative intensity of the diffraction peak at the respective position in the diffractogram.

(2) $$CrI=\frac{\left(I_{002}-I_{\text{am}}\right)}{I_{002}}\times 100\%$$

where *I*_002_ is the intensity of peak at a 2*θ* angle close to 22.5° and *I*_am_ is the scattering intensity of amorphous fraction at a 2*θ* angle close to 18°.

##### SEM Analysis

SEM was used to observe the morphology of the treated samples to follow the swelling effect. Poplar wood chips of 10 g (the number of samples of 10 g poplar chips is greater than 100) were immersed in the reaction system liquids at a solid‐liquid ratio of 1:10 and at room temperature for 12 h. The reaction systems are water, water with 7 wt% LiBr, FA and FA with 7 wt% LiBr, respectively. After soaking, the samples were freeze‐dried to remove residual solvents and maintain their original morphology. During SEM analysis, at least three samples from each reaction system were randomly selected for analysis.

##### Enzymatic Hydrolysis

Cellulose pulp and 20 FPU/g of cellulase were mixed in a 0.05 mol L^−1^ sodium acetate buffer (pH 4.8), resulting in a 2% substrate concentration. Then the enzymatic hydrolysis reaction was incubated at 50 °C, 120 r min^−1^ for 72 h. After enzymatic hydrolysis, 1 mL of the enzymatic hydrolysis solution was centrifuged to remove the pulp residue, and then it was denatured at 90 °C for 5 min. The supernatants were determined by HPLC to analyze the glucose content.

(3) $$\text{Sugar yield}\left(\%\right)=\frac{G}{G_{1}}\times 100$$

where *G*_1_ and *G* are the glucose amounts in pulp and hydrolysates, respectively.

##### Characterizations of Fractionated Components

The compositional analyzes of the cellulose pulp were conducted according to NREL laboratory protocols.^[^ ^58^ ^]^ The DP value is calculated based on the viscosity of the pulp, as shown in Equation (4). Pulp viscosity is measured according to the TAPPI T230 om‐99 standard. ≈150 to 200 mg of pulp is dissolved in copper ethylenediamine, and then the viscosity of the paper is characterized using CED and a capillary viscometer. The ash content of the pulp was determined following the TAPPI T221 om‐02 method. Roughly 2 g of the sample was first charred in a crucible over an electric hot plate until no visible smoke evolved. The crucible was then transferred to a muffle furnace maintained at 575 °C and ashed for 6 h. After cooling, the residue was weighed to calculate the ash content (Table S1, Supporting Information). The cellulose content of pulp is calculated by subtracting the mass of hemicellulose, lignin, and ash from the total mass. The content of ethanol‐toluene extract is neglected because it is almost always removed during fractionation in the organic acid system.

(4) $${\mathrm{DP}}^{0.905}=0.75\times [\eta ]$$

where *[η]* represents relative viscosity.

The content of hemicellulose was determined by an ICS‐5000 ion chromatography system (Thermo Fisher Scientific, MA, USA), equipped with a pulsed amperometric detector and a PA20 carbohydrate analysis column (3 × 150 mm). The oven and column temperatures were maintained at 30 °C, with the mobile phase comprising HPLC‐grade water, 250 mmol L^−1^ NaOH, 1 mmol L^−1^ NaAc, and an additional 50 mmol L^−1^ NaOH.

Glucose and xylose contents in the extracted solution were determined by HPLC equipped with an AminexHPX87H chromatographic column (300 × 7.8 mm, Bio‐Rad, USA) and a RID‐10 A differential refractive index detector. The analysis conditions were as follows: the mobile phase was a 0.005 mol L^−1^ H2SO4 solution, and the flow rate was 0.6 mL min^−1^.

The content of degradation products in extracted solution, such as acetic acid, furfural, and 5‐HMF, was determined according to the method published by Wang et al.^[^ ^37^ ^]^

##### Characterizations of Lignin

**FTIR spectroscopy:** The lignin samples were mixed with KBr in a dehydrated state. Subsequently, the mixture was pressed into pellets for FT‐IR detection. The spectra were scanned 16 times with a wavelength range of 4000–500 cm^−1^ and a resolution of 2 cm^−1^.^[^ ^59^ ^]^

**GPC analysis:** MWL, FA and LB‐FA lignin were analyzed on a Shimadzu Prominence‐i LC 2030 gel permeation chromatography system equipped with 2 tandem 300 × 7.8 mm (L × ID) Phenogel 5U columns (50 Å, 500 Å pores in series) protected with a 50 × 7.8 mm (L × ID) Phenogel 5U guard column (Phenomenex). An isocratic 100% tetrahydrofuran (HPLC grade without a stabilizer) was eluted at a flow rate of 1.0 mL min^−1^ and the column temperature was maintained at 35 °C and was detected using a variable wavelength detector at 280 nm. The apparent molecular weight was calibrated using polystyrene standards via detection at 254 nm.

**The content of Br in Lignin:** The bromine (Br) content in lignin was determined by ion chromatography (IC). Samples underwent oxygen combustion followed by purification through a solid‐phase extraction column, with a 400 μL injection volume into a Shimadzu HIC‐20 A SUPER IC system. Anion analysis utilized isocratic elution with a sodium carbonate (3.5 mm)/sodium bicarbonate (10 mm) mixture at 1.2 mL min^−1^, coupled with an anion suppressor, conductivity detector, and ultraviolet detector. Quantification of bromide was performed via a single‐point external standard method.

**NMR analysis:** Lignin analysis was conducted using an AVANCE II 500 spectrometer (Bruker, Karlsruhe, Germany). At room temperature, 150 mg of the lignin sample was dissolved in deuterated dimethyl sulfoxide (DMSO‐d6) and transferred to a 5‐mm NMR tube. The 2D HSQC NMR analysis was conducted using Bruker's standard pulse sequence, “hsqcetgpsi2". The measurement parameters included: at least 8 scans for ^1^H NMR with a scan width of 600 MHz, a collection time of 2.0 s, and a relaxation delay of 3.0 s; and at least 20 000 scans for ^13^C NMR with a scan width of 150 MHz, a collection time of 0.4 s, and a relaxation delay of 1.5 s. The content of various bonds in the lignin was determined by integrating the signals from the 2D HSQC NMR spectrum. A semi‐quantitative method was employed to integrate and calculate the signals.^[^ ^60^ ^]^

The analysis of lignin's ^31^P NMR spectra was conducted according to the method published in the ref.^[^ ^61^ ^]^ Lignin with 20 mg was dissolved in 500 μL of a mixture of anhydrous pyridine and deuterated chloroform (1.6:1, v/v). Subsequently, 100 μL of a cyclohexanol solution (10.85 mg mL^−1^ in anhydrous pyridine/CDCl_3_, 1.6:1 v/v) was added as an internal standard, and 100 μL of a chromium acetylacetonate solution (5 mg mL^−1^ in anhydrous pyridine/CDCl_3_, 1.6:1 v/v) was added as a relaxant. Lastly, 100 μL of phosphorylation reagent (2‐chloro‐4,4,5,5‐tetramethyl‐1,3,2‐dioxaphosphorocyclopentane, TMDP) was added, and the mixture was allowed to react for 15 min. The acquisition time and relaxation delay for the ^31^P NMR analysis were set to 1.5 and 2.0 s.

## Conflict of Interest

The authors declare no conflict of interest.

## Supporting information

Supplementary Material

## Data Availability

The data that support the findings of this study are available in the supplementary material of this article.
