# Supplementary material for: LiBr‐Formic Acid Enables Lignocellulosic Biomass Fractionation within 10 Minutes
Source: ChemSusChem. 2025 Oct 3;18(22):e202501354. doi: 10.1002/cssc.202501354 (PMC12642973; doi:10.1002/cssc.202501354)
Supplement: Supplementary file 1 — Supplementary Material [file CSSC-18-e202501354-s001.pdf]

Supplementary Information

LiBr-Formic Acid Enables Lignocellulosic Biomass Fractionation Within 10 Minutes

Qi Bu [1], Shuzhen Ni[1], Zhaojiang Wang[1], Yingjuan Fu[1], Yongchao Zhang*[1], Wenyang Xu*[2]

[1] State Key Laboratory of Green Papermaking and Resource Recycling, Qilu University of Technology, Shandong Academy of Sciences, Jinan 250353, Shandong, China.

[2] Max Planck Institute of Colloids and Interfaces, Science Park Golm, 14476 Potsdam, Germany.

* Corresponding author: yczhang@qlu.edu.cn (Y. Zhang); wenyang.xu@mpikg.mpg.de (W. Xu).

Supporting Figures and Tables

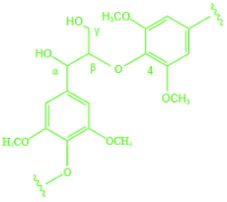

A

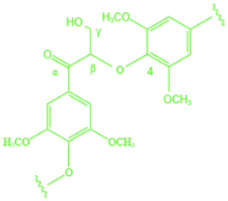

A'

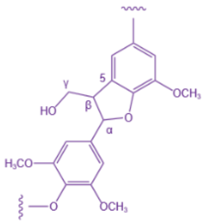

B

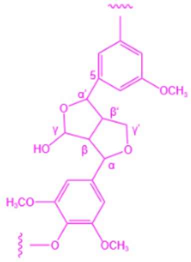

C

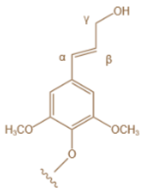

I

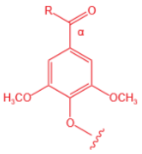

S

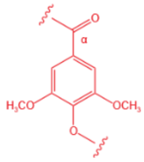

S'

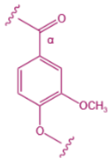

G

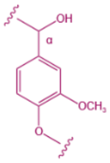

G'

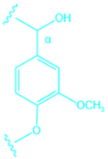

H

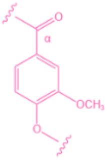

PB

Figure S1

A, β-O-4 aryl ether linkage; A', β-O-4 aryl ether linkage with oxidation at the Cα position; B, resinol (β-β) substructure; C, phenylcoumaran substructure β-5; G, guaiacyl unit; G', Cα-oxidized guaiacyl unit; S, syringyl unit; S', Cα-oxidized syringyl unit; H, p-hydroxyphenyl unit; PB, p-hydroxybenzoate structure; I, p-hydroxycinnamyl end group.

Table S1. The ash content of LB-FA and FA's cellulose pulp

Reaction condition	LB-FA	Reaction condition	FA
90 ℃, 10 min, 7wt% LiBr	0.11%	90 ℃, 10 min	0.41%
100 ℃, 10 min, 7wt% LiBr	0.08%	100 ℃, 10 min	0.14%
110 ℃, 10 min, 7wt% LiBr	0.07%	110 ℃, 10 min	0.11%
120 ℃, 10 min, 7wt% LiBr	0.04%	120 ℃, 10 min	0.09%
120 ℃, 0 min, 7wt% LiBr	0.09%	120 ℃, 0 min	0.15%
120 ℃, 5 min, 7wt% LiBr	0.05%	120 ℃, 5 min	0.1%
120 ℃, 10 min, 7wt% LiBr	0.04%	120 ℃, 10 min	0.09%
120 ℃, 15 min, 7wt% LiBr	0.04%	120 ℃, 15 min	0.08%
120 ℃, 10 min, 1wt% LiBr	0.08%	-	-
120 ℃, 10 min, 3wt% LiBr	0.08%	-	-
120 ℃, 10 min, 5wt% LiBr	0.05%	-	-
120 ℃, 10 min, 7wt% LiBr	0.04%	-	-

Table S2. The crystallinity index (*CrI*) and DP of LB-FA cellulose pulp

	CrI (%)	DP
Poplar powder	48.3	-
FA pulp	66.0	682
LB-FA pulp	69.4	674

Table S3. Carbohydrate content in immersion liquid

	Glucose mg/L	Xylose mg/L	Arabinose mg/L
Water	45.2	11.4	ND
LiBr-Water	55.3	19.9	ND
FA	93.2	132.9	5.4
LB-FA	150.3	215.3	100.4

ND: Not detected

Table S4 Signal attributions corresponding to major lignin structural units in 2D HSQC profiles

Signal	δ_C/δ_H(ppm)	Structural units
C_β	51.9/3.6	Cβ-Hβ in phenylcoumarane substructures (C)
B_β	54.2/3.1	Cβ-Hβ in resinol substructures (B)
MeO	56.0/3.7	C-H in methoxyls
C_γ	62.8/3.7	Cγ-Hγ in phenylcoumaran substructures (C)
A_γ	59.7/3.5	Cγ-Hγ in β-O-4’ substructures (A)
A_γ’	63.5/4.5	Cγ-Hγ in γ-acylated β-O-4’ substructures (A’)
A_α	71.9/4.8	Cα-Hα in β-O-4’ substructures（A）
$A_{\beta(G)}$	83.9/4.3	Cβ-Hβ in β-O-4’ substructures linked to G
B_α	85.0/4.7	Cα-Hα in resinol substructures (B)
$A_{\beta(S)}$	86.3/4.1	Cβ-Hβ in β-O-4 substructures linked to S units (A)
C_α	87.5/5.5	Cα-Hα in phenylcoumaran substructures (C)
$S_{2,6}$	104.5/6.6	C2,6-H2,6 in etherified syringyl units (S)
$S_{2,6}$'	106.0/7.2	C2,6-H2,6 in oxidized (Cα=O) syringyl units (S’)
G_2	110.9/6.9	C2-H2 in guaiacyl units (G)
G_5	114.9/6.7	C2-H2 in guaiacyl unit (G)

Signal	δ_C/δ_H(ppm)	Structural units
G_6	118.9/6.8	C6-H6 in guaiacyl units (G)
$H_{2,6}$	128.2/7.2	C2,6-H2,6 in p-hydroxyphenyl units (H)
$PB_{2,6}$	131.3/7.6	C2,6-H2,6 in p-hydroxybenzoate substructures (PB)
X_5	62.8/3.2	C5-H5 in *β*-D-xylopyranoside
X_2	70.1/3.2	C2-H2 in *β*-D-xylopyranoside
X_3	73.5/3.3	C3-H3 in *β*-D-xylopyranoside
X_4	76.0/3.5	C4-H4 in *β*-D-xylopyranoside

Table S5 Molecular weight distribution of MWL, FA, and LB-FA lignin

Sample	M_w	M_n	PDI (M_w/M_n)
MWL	6945	4699	1.5
FA	9327	2895	3.2
LB-FA	8565	796	10.8

Table S6 FA and LB-FA Bromine content in lignin

Sample	Br content (mg/g)
FA lignin	17.6
LB-FA lignin	39.4

Table S7 Comparisons of the performance among different pretreatment/fractionation processes in terms of removal of hemicelluloses and lignin as well as enzyme hydrolysis efficiency

Process	Biomass	Fractionation condition	Xylan; lignin removal (%)	Enzyme loading [a]	Sugar yield (%) at 12 h	Sugar yield (%)	Reference
This work	40-60 mesh Poplar wood	T=120 ℃; t=10 min	~100；96	CTec3=20	＞90	95.9	
ethanol	Sugarcane bagasse	50% ethanol + Kraft green liquor<br>T=80 ℃；t=3 h	N[b]; 52.1	Cellulase=18	＜30	65.1	[1]
		T=100 ℃；t=3 h	N; 70.8		＜50	79.3	
		T=140 ℃；t=3 h	24.1; 89.7		＜60	97.9	
		T=160 ℃；t=3 h	N; 95.3		＜70	~97	

Acetone	Birch particles	T=140 ℃; t=2 h<br>50% w/w aqueous acetone and H_2SO_4	92; 86	Accelerase TRIO =10 FPU/ g solids (=14)	＜60	~90	[2]
Dilute acid	20-40 mesh Wheat straw	T =120 ◦C; t =1 h<br>0.5% H_2SO_4	46.7; 3.7	CTec2=21.7	＜20	28.8	[3]
		1% H_2SO_4	70.7; 4.7	CTec2=19.9	＜30	44.3	
		3% H_2SO_4	86.1; 1.7	CTec2=17.7	＜30	45.9	
		T =75 ◦C; t =3 h<br>8% H_2SO_4 + $NaClO_2$	94.5; 1.8	CTec2=17.2	＜40	52.6	
Formic acid	20-80 mesh Poplar wood	88% formic acid<br>T=80 ℃; t=6 h	79.6; 62.5	CTec2 =20	＜60	＞90	[4]
	20-80 mesh Chinese fir	T=80 ℃; t=6 h	65.8; 35.1	CTec2 =20	＜40	＜60	

	20-80 mesh Bamboo	T=80 ℃; t=6 h	70.9; 12.7	CTec2 =20	＜80	＞95	
	Wheat straw	T=90 ℃; t=6 h	65.2; 14.8	CTec2 =20	＜80	＞95	
	40-60 mesh Poplar wood	T=90 ℃；t=3 h<br>30% formic acid<br>5%methanesulfonic acid	~86.3; 20.4	CTec3 =50	＜40	45.9	[5]
AHF (*p*-TsOH)	Switch grass	T =80 ◦C; t =1.5 h<br>60 wt% *p*-TsOH	57.9; 76.4	CTec3 =10	＜20	~54	[6]
	Poplar NE222	T =65 ◦C; t =35 min<br>75 wt% *p*-TsOH	76.1; 77.7	CTec3 =20	＜40	＞90	[7]
		T =65 ◦C; t =1 h<br>75 wt% *p*-TsOH	80.8; 82.1		＜40	＞90	
MAHF (MA)	Wheat straw	T=80 ℃；t=2 h	65.6; 37.8	CTec3 =10	N	41.6	[8]

		Maleic acid =40 wt%					
		T=100 ℃；t=30 min<br>Maleic acid =60 wt%	67.2; 53.6		N	77.7	
		T=120 ℃；t=1 h<br>Maleic acid =60 wt%	76.1; 56.6		N	89.4	
	Switch grass	T=120 ℃；t=30 min<br>Maleic acid =60 wt%	79; 57.8		＜20	~78	[6]
DES	40-60 mesh Poplar wood	T=110 ℃；t=6 h<br>ChCl: lactic acid: salicylic acid =1:4:1;	91.2; 75.3	CTec3 =20	＜50	82.7	[9]
	Bamboo	T=130 ℃；t=10 min<br>ChCl: 1,4-BDO: $AlCl_3$= 25:50:1	84.2; 61.3	CTec2 =25	N	89.6	[10]

IL	Wheat straw	T=131 ℃；t=88 min<br>41.3 wt% [emin][HSO4]<br>a direct enzymatic hydrolysis of the reaction solid	79.6; ~0	CTec2≈20	N	75.8	[11]
		Enzymatic hydrolysis after lignin removal			N	91.3	

[a] Unit in FPU/ (g substrate glucan) unless indicated; [b] No data provided.

Reference

[1] Z. Zhou, W. Xue, F. Lei, Y. Cheng, J. Jiang, D. Sun, *Ind. Crop. Prod.* **2016**, *90*, 100–109.

[2] A. Smit, W. Huijgen, *Green Chem.* **2017**, *19*, 5505–5514.

[3] H. Chen, X. Zhao, D. Liu, *ACS Sustain. Chem. Eng.* **2016**, *4*, 6668–6679.

[4] H. Qiao, Y. Wang, Z. Ma, M. Han, Z. Zheng, J. Ouyang, *Bioresour. Technol.* **2023**, *374*, 128747.

[5] R. Wu, Y. Li, X. Wang, Y. Fu, M. Qin, Y. Zhang, *Bioresour. Technol.* **2023**, *369*, 128410.

[6] C. Su, K. Hirth, Z. Liu, Y. Cao, J. Y. Zhu, *Ind. Crop. Prod.* **2021**, *159*, 113017.

[7] L. H. Chen, J. Z. Dou, Q. L. Ma, N. Li, R. C. Wu, H. Y. Bian, D. J. Yelle, T. Vuorinen, S. Y. Fu, X. J. Pan, J. Y. Zhu, *Sci. Adv.* **2017**, *3*, e1701735.

[8] C. Su, K. Hirth, Z. Liu, Y. Cao, J. Y. Zhu, *GCB Bioenergy* **2021**, *13*, 1407–1424.

[9] H. Zhang, R. Wu, X. Chen, S. Ni, C. Xu, Y. Fu, M. Qin, Y. Zhang, *Ind. Crop. Prod.* **2025**, *225*, 120489.

[10] J. Cheng, X. Liu, Y. Zhan, J. Wang, X. Meng, X. Zhou, C. G. Yoo, C. Huang, C. Huang, G. Fang, A. J. Ragauskas, *ChemSusChem* **2024**, *17*, DOI 10.1002/cssc.202301161.

[11] A. M. da Costa Lopes, R. M. G. Lins, R. A. Rebelo, R. M. Lukasik, *Green Chem.* **2018**, *20*, 4043–4057.
